# Novel Compound Heterozygous Mutation in *TRAPPC9* Gene: The Relevance of Whole Genome Sequencing

**DOI:** 10.3390/genes12040557

**Published:** 2021-04-12

**Authors:** Maria Isabel Alvarez-Mora, Jordi Corominas, Christian Gilissen, Aurora Sanchez, Irene Madrigal, Laia Rodriguez-Revenga

**Affiliations:** 1 Department of Biochemistry and Molecular Genetics, Hospital Clinic, 08036 Barcelona, Spain; ASANCHEZ@clinic.cat (A.S.); imadbajo@clinic.cat (I.M.); lbodi@clinic.cat (L.R.-R.); 2 Department of Human Genetics, Radboud UMC, 6525 GA Nijmegen, The Netherlands; Jordi.CorominasGalbany@radboudumc.nl (J.C.); christian.gilissen@radboudumc.nl (C.G.); 3 Institut d’Investigacions Biomèdiques August Pi i Sunyer (IDIBAPS) and CIBER of Rare Diseases (CIBERER), 08036 Barcelona, Spain

**Keywords:** *TRAPPC9*, whole genome sequencing, neurodevelopmental disorders, neuropsychiatric disorders, compound heterozygous mutations, missense mutation, structural variants

## Abstract

Advances in high-throughput technologies and its implementation worldwide have had a considerable impact on the elucidation of the molecular causes underlying neurodevelopmental psychiatric disorders, especially for autism spectrum disorder and intellectual disability (ID). Nevertheless, etiology remains elusive in close to 50% of cases, even in those families with multiple affected individuals, strongly hinting at a genetic cause. Here we present a case report of two siblings affected with severe ID and other comorbidities, who embarked on a genetic testing odyssey until diagnosis was reached by using whole genome sequencing (WGS). WGS identified a maternally inherited novel missense variant (NM_031466.7:c.1037G > A; p.Gly346Glu) and a paternally inherited 90 kb intragenic deletion in *TRAPPC9* gene. This report demonstrates the clinical utility of WGS in patients who remain undiagnosed after whole exome sequencing.

## 1. Introduction

Neurodevelopmental psychiatric disorders, including autism spectrum disorder (ASD), intellectual disability (ID), epilepsy, and schizophrenia (SZ), are a group of heterogeneous disorders associated mainly with the disruption of the tightly coordinated events that lead to brain development [1]. This process results from highly complex and coordinated activity involving genetic and environmental processes. This group of disorders constitutes a serious health problem in our society, accounting as a group for one of the top 30 leading contributors to overall disease burden, as measured by global disability adjusted life years [2].

Neurodevelopmental disorders (NDDs) are associated with a sex bias, with a male:female ratio of 2:1 existing among individuals with ID and a 4:1 ratio for individuals with ASD [3]. NDDs are clinically heterogeneous, with overlapping symptoms, and frequently co-occur, suggesting a common genetic etiology; this explains the high degree of comorbidity among them [4]. Much like their phenotypes, the genetic etiology underlying NDDs is highly heterogeneous, with varying degrees of genetic overlap and penetrance, or expressivity, across phenotypes. Many studies have suggested shared molecular pathways for ID and other NDDs. This has been inspired by the high comorbidity that is commonly observed between ID and other cognitive impairments such as ASD and epilepsy [5]. Insights from “The Psychiatric Cell Map Initiative” have evidenced three main molecular pathways involved in these disorders: protein synthesis, transcriptional or epigenetic regulation and synaptic signaling [6,7].

Advances in high-throughput technologies and their implementation worldwide have had a considerable impact on elucidating the molecular causes underlying NDDs, especially for ID and ASD [5]. The introduction of whole exome sequencing (WES) into medical practice has transformed the diagnosis and management of patients with genetic disease. Nevertheless, etiology remains elusive in close to 50% of NDD cases, even in those families with multiple affected individuals, strongly hinting at a genetic cause. A step forward is whole genome sequencing (WGS), which delivers a base-by-base view of all genomic alterations, including single nucleotide variants (SNV), insertions and deletions, copy number variations (CNVs) and structural variations (SVs). The combination of emerging short-read and long-range genome sequencing has greatly improved the identification of this last type of genetic variation. SV represents the greatest source of genetic diversity in the human genome [8]. Therefore, it is not surprising that both de novo and inherited SVs are frequently linked to the pathogenesis of NDDs such as ASD, ID, SZ and developmental delay. However, the overall contribution of SV effects in disease etiology is still unclear [9].

Here we present a case report of a two siblings affected with severe ID and other comorbidities, who embarked on a genetic testing odyssey of more than ten years before diagnosis was reached by applying WGS with two distinct analysis pipelines.

## 2. Material and Methods

### 2.1. Case Report

**Figure 1:**
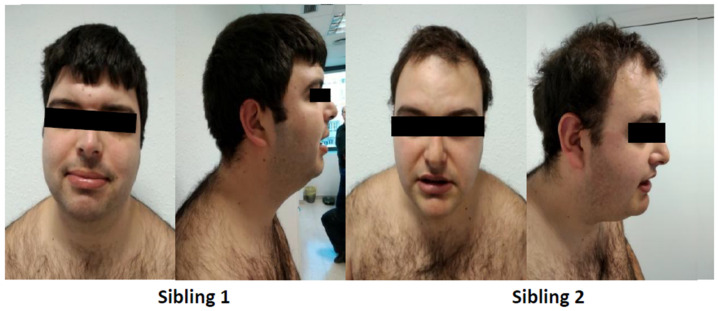
Images of patients carrying heterozygous mutation in *TRAPPC9* gene.

The family consisted of two affected siblings and a non-affected daughter born from a non-consanguineous Spanish family. This family was referred in 2007 to the Clinical Genetics consultation of the Hospital Clinic of Barcelona (Barcelona, Spain). Both siblings were characterized by severe ID, absent speech, behavioral abnormalities such as “happy disposition”, slight obesity and mild facial dysmorphism with mild myopia (Figure 1). Brain magnetic resonance imaging showed dysgenesis of the *corpus callosum* and *cisterna magna*. Standard karyotyping, CGG repeat expansion in *FMR1 gene*, MLPA (Salsa P036 and P070, MRC-Holland, Amsterdam, The Netherlands), array-CGH (4 × 44K, Agilent Technologies, Santa Clara, CA, USA) and WES testing were normal (Agilent’s V3 capture kit, Agilent Technologies, and sequenced on a HiSeq™ 2000 Sequencing System, Illumina, San Diego, CA, USA).

All individuals provided written consent, and the study was approved by the Institutional Review Boards of the Hospital Clinic of Barcelona.

### 2.2. Whole Genome Sequencing and Data Analysis

WGS was performed to individual II.2 in Macrogen Inc. (Macrogen, Seoul, Korea) as a part of a research project (COHORTES/Programa de Enfermedades Raras No Diagnosticadas). Data analysis was performed in the Genome Diagnostics Nijmegen of the Radboud University Medical Center (Nijmegen, The Netherlands). Alignment against the GRCh37 human reference genome was performed with a Burrows-Wheeler Aligner (BWA) v.0.7.8 [10]. PCR duplicates were marked using Samtools v.1.5. [11] Variant calling was carried out using xAtlas v.0.1 and variants were annotated using an *in-house* developed pipeline. This variant annotation was performed using the Variant Effect Predictor (VEP V.91) [12] and Gencode V.34lift37 basic gene annotations. Frequency information was added from GnomAD V.2.1.1. Allelic variants with frequency >0.01 in any of the databases used (GnomAD (https://gnomad.broadinstitute.org/; accessed on 21 July 2018), ExAC (http://exac.broadinstitute.org/; accessed on 21 July 2018) and 1000Genomes (https://www.internationalgenome.org/; accessed on 21 July 2018)) were discarded. Data analysis was focused on missense, nonsense, frameshift, and small insertion/deletion variants. CNVs were called using Control-FREEC v9.1 [13]. SVs were called using Manta Structural Variant Caller V.1.1.0 (Illumina), which uses a paired end and split read evidence approach for SV identification [14]. SVs and CNVs were annotated using an *in-house* developed pipeline. This pipeline was based on ANNOVAR and Gencode V.34lift37 basic gene annotations. Additional frequency information was added from GnomAD V.2.1, 1000G V.8 and GoNL SV release 1 databases.

### 2.3. Segregation Studies

Segregation analyses of *Trafficking protein particle complex 9* (*TRAPPC9*) variants were performed in available relatives. Segregation of the SNV was performed by Sanger sequencing. PCR primers were designed using the Primer3 Input version 4.0.0 web tool (http://primer3.ut.ee/; accessed on 23 August 2018). PCR products were directly sequenced using the BigDye^®^ Terminator version 3.1 Cycle Sequencing Kit (Applied Biosystems, Foster City, CA, USA). The reaction was run in an ABI Prism 3100XL automated sequencer (Applied Biosystems, Foster City, CA, USA) and the results were analyzed with SEQUENCE^®^ Pilot version 4.0.1 software (JSI medical systemsCorp, New York, NY, USA). Segregation of the SV was performed using a high resolution CGH-microarray (60K) following the manufacturer’s recommendations (qGenomics, Barcelona, Spain). Data was analyzed with the qGenviewer Software v2.1.1 (qGenomics).

## 3. Results

**Figure 2:**
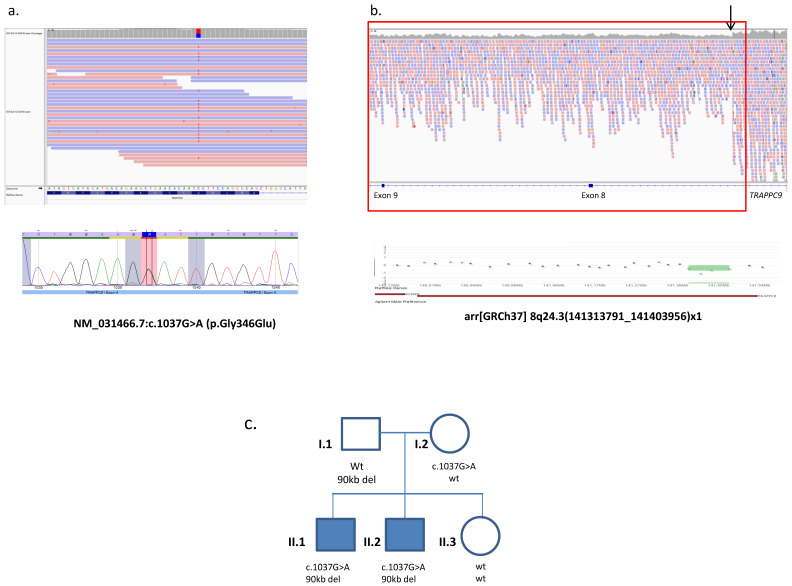
Molecular analysis of *TRAPPC9* mutations and segregation analyses. (**a**) Upper: view of the missense variant in heterozygosity in *TRAPPC9* gene in the Integrative Genome Viewer; lower: validation of the variant by Sanger sequencing. (**b**) Upper: screenshot of the Integrative Genome Viewer showing the structural variant in heterozygosity (red square) removing exons 8 and 9 in *TRAPPC9* (blue boxes in the bottom). Note the decreased coverage in the deleted region (red vertical arrows). The size of the alteration does not allow for capturing of the whole deletion; lower: validation of the intragenic deletion by microarrayCGH (60K). (**c**) Pedigree of the family and results of segregation analysis. Affected individuals are shown as shaded squares.

Genetic investigation performed prior to WGS failed to identify the genetic alteration explaining the disease in this family. Following the guideline recommendations for the study of ID in 2007, the application of conventional karyotype, analysis for fragile X syndrome and subtelomeric rearrangements did not identify pathogenic variants in the affected individuals. After CGH microarray implementation, CNVs were discarded by microarrayCGH (44K). In addition, WES was performed on both siblings as previously described [15]. Data analysis of the variants shared by both patients evidenced the presence of a novel missense variant in heterozygosis in *TRAPPC9* gene (NM_031466.7; chr8 (GRCh37): g.141445327 C > T; c.1037G > A; p.Gly346Glu). This variant was not present in the public databases and was predicted to be deleterious by nine different programs. At this time, pathogenic variants in *TRAPPC9* gene were exclusively associated to consanguineous families with homozygous loss-of-function (LOF) variants. Since no second mutation was found on the other *TRAPPC9* allele, and CNVs were previously discarded by array-CGH (44K), this variant was finally classified as a variant of unknown significance (VUS). Next, WGS was performed on individual II.2, and genetic diagnosis was reached combining two WGS data analysis pipelines: the one used for calling SNVs and indels variants and the one used for calling SV. The first pipeline confirmed the presence of the previously detected variant in *TRAPPC9* gene (Figure 2a), and data analysis for SV detected a novel 90 kb intragenic deletion spanning from exon 8 and 9 in *TRAPPC9* gene (Figure 2b). Although this was a novel deletion, intragenic deletions of *TRAPPC9* gene were recently described as pathogenic variants as detected in six patients with ID [16]. The deletion was confirmed by a higher resolution microarrayCGH (60K), and the breakpoints of the SV were redefined to arr[GRCh37] arr8q24.3(141313791_141403956) × 1 (Figure 2b).

The clinical presentation of the patients was compatible with the OMIM description of an autosomal recessive ID disorder mapping to the short arm of chromosome 8 (Mental retardation autosomal recessive 13; OMIM # 613192), and molecular analysis of relatives verified the segregation of these variants with the disease in the family. Sanger sequencing revealed that the missense variant was maternally inherited, and microarrayCGH identified the intragenic deletion in the father. The unaffected sister did not present any of the identified variants (Figure 2c).

## 4. Discussion

Mutations in TRAPP proteins have been collectively termed “TRAPPopathies” [17]. *TRAPPC9* gene encodes a subunit of the trafficking protein particle II (TRAPPII), one out of three TRAPP complexes that act as multimeric guanine nucleotide exchange factors (GEFs) to activate certain GTPases, helping regulate vesicular trafficking between organelles. The TRAPP complex acts as an activator of a subgroup of Ypt/RAB GTPases required for secretion and macroautophagy/autophagy. TRAPP subunits are conserved from yeast to human cells and have been largely implicated in human disease [18].

TRAPPC9, also known as NIK-and-IKK2-binding protein (NIBP), is extensively expressed in the nervous system and plays a role in regulating neurogenesis and neuronal differentiation. It plays a role in both the regulation of protein trafficking and the neuronal NF-kB signaling pathway [19,20]. In the former, TRAPPC9 is involved in the trafficking of cargo from the endoplasmic reticulum to the Golgi; interestingly, impairment of vesicular trafficking has been observed as a common biologic defect in neurologic disorders [19]. In addition, TRAPPC9 is involved in the activation of NFkB, which remains sequestered in the cytoplasm while is not activated [20]. The NFkB signaling pathway is involved in the regulation of many different cellular pathways, including memory, neurogenesis, and synaptic plasticity [21]. Although the mechanisms underlying how the loss of TRAPPC9 impairs brain development and function are yet to be defined, it is that possible these two roles for TRAPPC9 might contribute to neuronal impairment. In this regard, Ke and collaborators (2020) recently reported a *Trappc9* knock-out mouse that recapitulated features of human ID [22]. They demonstrated in the mouse model that *Trappc9* deficiency impairs learning and memory by causing imbalance of dopamine D1 and D2 neurons [22].

**Figure 3:**
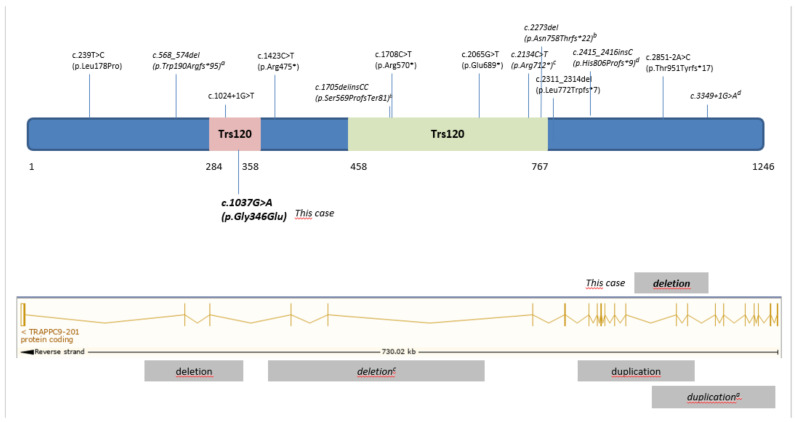
Summary of reported *TRAPPC9* mutations. Graphical representation of TRAPPC9 protein based on the longest isoform that encodes for 1246 amino acids (ENST00000389328.4/NM_031466). All mutations were previously reported in homozygous changes, except for three heterozygous changes, which are presented in italics.

In humans, pathogenic variants in *TRAPPC9* gene are associated with a nonsyndromic form of ID named “Mental retardation, autosomal recessive 13” (OMIM # 613192), although recurrent brain abnormalities and obesity are commonly observed in some patients. The delineation of the phenotype was made based on multiplex consanguineous families from different ethnic backgrounds that carried homozygous LOF variants, including frameshift, nonsense and splicing mutations as well as intragenic deletions and duplications. To date, only three families with compound heterozygous disease LOF variants have been reported (Figure 3). In 2018, Mortreux and collaborators reported the first two non-consanguineous families who were compound heterozygotes for an intragenic CNV and a LOF variant [16]. Shortly after, a novel compound heterozygous family was reported harboring a frameshift variant and a splice site mutation (c.3349 + 1G > A) [23]. A recently report found a non-consanguineous family with a paternal LOF frameshift variant and a maternal complex allele with a missense c.1705T > C (p.Ser569Pro) and a frameshift variant (c.1708dupC, p.Arg570Profs*80) [24]. However, this maternal allele certainly corresponds to an indel mutation c.1705delinsCC (p.Ser569ProfsTer81) instead of a complex allele. To our knowledge, there is only one pathogenic homozygous missense variant associated with this syndrome. The pathogenic missense c.533T > C (p.Leu178Pro) variant was identified in two siblings with severe ID, microcephaly and hypoplasia of the corpus callosum [25]. Here, we report for the first time a non-consanguineous family with compound heterozygous mutation consisting of a novel missense variant and an intragenic deletion in *TRAPPC9* gene. The missense mutation p.Gly346Glu identified in our family is located in the Trs120 region of the TRAPPC9 protein (Figure 3), which is considered a relevant region for the stability of TRAPP complexes [26]. This report extends the mutational spectrum of *TRAPPC9* mutations since, irrespective of the type of disease causing the variant, all patients are characterized by severe ID, behavioral abnormalities, absent speech and white matter abnormalities.

Furthermore, genetic alteration of *TRAPPC9* gene has not only been associated with ID but also with SZ [27] and attention deficit/hyperactivity disorder [28], and it is currently considered as a strong candidate risk gene for ASD by The Simons Foundation Autism Research Initiative (https://gene.sfari.org/database/human-gene/TRAPPC9; accessed on 1 March 2021). It has recently been suggested that heterozygous LOF of *TRAPPC9* might be a risk factor for ASD, which would be further exacerbated in cases with homozygous or compound heterozygous mutations [20].

## 5. Conclusions

In the last years, WES has emerged as a comprehensive and cost-effective approach to identify pathogenic variants in the protein-coding regions of the genome. However, the high percentage of undiagnosed cases in NDDs suggests that there is a large number of disease causing variants that are not being captured by the current approaches. This scenario might be comparable to the parable of the Blind Men and the Elephant, trying to reconstruct the complexity of human disease through fragmented experience; thus, we argue that WGS offers a promising alternative for undiagnosed patients.

For many years, the cost of sequencing an entire genome remained prohibitively expensive for routine use in clinical practice. However the significant decline in sequencing costs will lead to the implementation of WGS as a single genetic test to reliably identify and characterize the comprehensive spectrum of genetic variation, thus increasing the diagnostic yield of patients affected with a neurodevelopmental/neuropsychiatric disorder.

Although the characterization of functional non-coding variants remains challenging, efforts should be focused on the most well-known relevant regulatory regions, including the 3′ and 5′ untranslated regions and the putative noncoding regulatory DNA corresponding to promoters and enhancers and the transcription factor-binding sites [29,30,31]. However, our report supports the fact that the greatest advantage of WGS is the more accurate detection of CNV and SV rather than the discovery of disease causing variants in non-coding regions of the human genome. Notwithstanding that the implementation of WGS in routine genetic diagnosis is still challenging, this report demonstrates the clinical utility of WGS for individuals in whom WES fails to identify a pathogenic variant.

## Data Availability

Whole genome sequencing data reported is available in the NCBI Sequence Read Archive under accession number PRJNA603428 (SRA; https://www.ncbi.nlm.nih.gov/sra/PRJNA603428; date of submission: 31 March 2020).
